# Supplementary material for: The Evolution and Characterization of the RNA Interference Pathways in Lophotrochozoa
Source: Genome Biol Evol. 2024 May 7;16(5):evae098. doi: 10.1093/gbe/evae098 (PMC11114477; doi:10.1093/gbe/evae098)
Supplement: evae098_Supplementary_Data [file evae098_supplementary_data.zip › Supplementary_materials.zip!/selected_miRNAs/NoGe_selected_miRNAs/mirdeep_pdf_files/NMRB01002591.1_Notospermus_geniculatus_strain_Ushimado_scaffold2591,_whole_genome_shotgun_sequence_39375.pdf]

| | | |
|---|---|---|
| Provisional ID | : | NMRB01002591.1_Notospermus_geniculatus_strain_Ushimado_scaffold2591,_whole_genome_shotgun_sequence_39375 |
| Score total | : | 55280.5 |
| Score for star read(s) | : | 3.9 |
| Score for read counts | : | 55271.8 |
| Score for mfe | : | 1.8 |
| Score for randfold | : | 0 |
| Score for cons. seed | : | 3 |
| Total read count | : | 108425 |
| Mature read count | : | 108131 |
| Loop read count | : | 0 |
| Star read count | : | 294 |

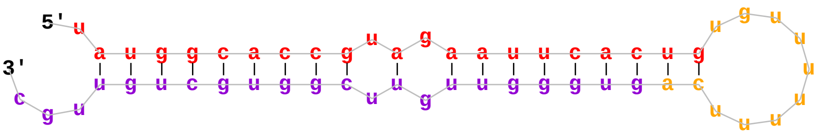


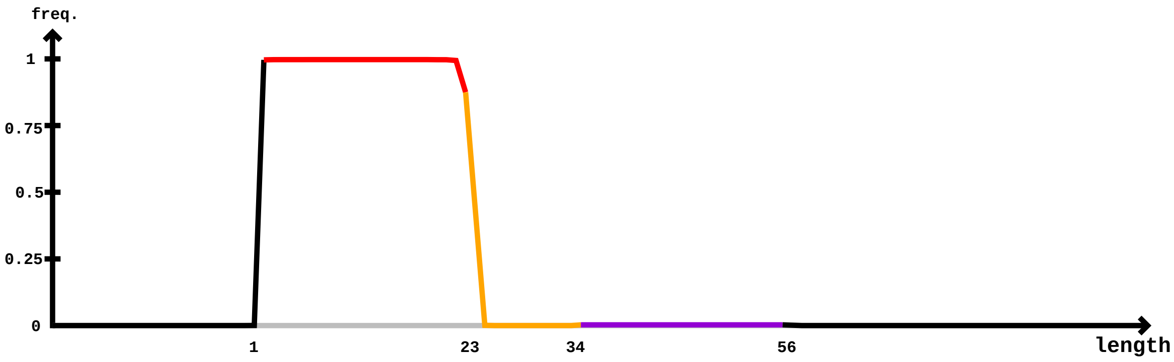


**Mature** **Star**

```
5'- ucaucucugcuuacaagagcuauggcaccguagaauucacuguguuuuuuucagugggguuguucggugcuguugcuuuugauguagauagcugcuguugucacuugugauuc   -3'    obs
    ucaucucugcuuacaagagcuauggcaccguagaauucacuguguuuuuuucagugggguuguucggugcuguugcuuuugauguagauagcugcuguugucacuugugauuc          exp
    .......(((.(.(((((((.(((((((((.(.(((((((((.........))))))))).).))))))))).)))))))).)))((((((....))))))...........   reads   mm   sample
    ....................uauggcaccguagaauuc..........................................................................   3       0    Mm3
    ....................uauggcaccguagaauuca.........................................................................   3       0    Mm3
    ....................uauggcaccguagaauucac........................................................................   36      0    Mm3
    ....................uauggcaccguagaauAcacu.......................................................................   2       1    Mm3
    ....................uauggcaccguagaauCcacu.......................................................................   1       1    Mm3
    ....................uauggcaccguGgaauucacu.......................................................................   1       1    Mm3
    ....................uauggcaccguagaauuUacu.......................................................................   5       1    Mm3
    ....................uauggcaccguagaauucacG.......................................................................   3       1    Mm3
    ....................uauggcaccgCagaauucacu.......................................................................   1       1    Mm3
    ....................uauggcaccguagaauuAacu.......................................................................   3       1    Mm3
    ....................uauggcaccUuagaauucacu.......................................................................   1       1    Mm3
    ....................uauggcaccguagGauucacu.......................................................................   1       1    Mm3
    ....................uauggcGccguagaauucacu.......................................................................   2       1    Mm3
    ....................uauggcaccguagaauucacA.......................................................................   2       1    Mm3
    ....................uauggUaccguagaauucacu.......................................................................   1       1    Mm3
    ....................uauggcaccguagaauucacu.......................................................................   783     0    Mm3
    ....................uauggcUccguagaauucacug......................................................................   1       1    Mm3
    ....................uauggUaccguagaauucacug......................................................................   1       1    Mm3
    ....................uauggcacUguagaauucacug......................................................................   4       1    Mm3
    ....................uaugUcaccguagaauucacug......................................................................   1       1    Mm3
    ....................uauggcaccguaUaauucacug......................................................................   1       1    Mm3
    ....................uauggcaccguagaauucacuU......................................................................   4       1    Mm3
    ....................uauggcaccguagaauucacuA......................................................................   1       1    Mm3
    ....................uauggNaccguagaauucacug......................................................................   1       1    Mm3
    ....................uauggcaccguagaauuAacug......................................................................   28      1    Mm3
    ....................uauggcaccguCgaauucacug......................................................................   4       1    Mm3
    ....................uauggcaccguagaauuUacug......................................................................   15      1    Mm3
    ....................uauggcaccguGgaauucacug......................................................................   2       1    Mm3
    ....................uauggcaccguagaCuucacug......................................................................   1       1    Mm3
    ....................uauggcaccguagaauGcacug......................................................................   6       1    Mm3
    ....................uauggcGccguagaauucacug......................................................................   2       1    Mm3
    ....................uauggcaccguUgaauucacug......................................................................   1       1    Mm3
    ....................uauggcaccgCagaauucacug......................................................................   4       1    Mm3
```

Mature Star

| | | | |
|---|---|---|---|
| ucaucucugcuuacaagagcuauggcaccguagaauucacuguguuuuuuucaguggguuguucggugcuguugcuuuugauguagauagcugcuguugucacuugugauuc | | | |
| ....................uauggcaUcguagaauucacug...................................................................... | 1 | 1 | Mm3 |
| ....................uauggcaccguagaauCcacug...................................................................... | 10 | 1 | Mm3 |
| ....................uauggcaccguagaauucacCg...................................................................... | 1 | 1 | Mm3 |
| ....................uauUgcaccguagaauucacug...................................................................... | 1 | 1 | Mm3 |
| ....................uauggcaccguagaGuucacug...................................................................... | 1 | 1 | Mm3 |
| ....................uauggcaccUuagaauucacug...................................................................... | 1 | 1 | Mm3 |
| ....................uauAgcaccguagaauucacug...................................................................... | 3 | 1 | Mm3 |
| ....................uauggcaccgGagaauucacug...................................................................... | 6 | 1 | Mm3 |
| ....................uauggcNccguagaauucacug...................................................................... | 1 | 1 | Mm3 |
| ....................uauggcaccguagaauAcacug...................................................................... | 8 | 1 | Mm3 |
| ....................uauggcaccguagaUuucacug...................................................................... | 1 | 1 | Mm3 |
| ....................Nauggcaccguagaauucacug...................................................................... | 1 | 1 | Mm3 |
| ....................uauggcaccAuagaauucacug...................................................................... | 3 | 1 | Mm3 |
| ....................uauggcaccguagaauucacug...................................................................... | 2731 | 0 | Mm3 |
| ....................uauggcaccguagaauuGacug...................................................................... | 10 | 1 | Mm3 |
| ....................uGuggcaccguagaauucacug...................................................................... | 2 | 1 | Mm3 |
| ....................uUuggcaccguagaauucacug...................................................................... | 1 | 1 | Mm3 |
| ....................uauggcaccgAagaauucacug...................................................................... | 9 | 1 | Mm3 |
| ....................uauggcaccguagaauucacGg...................................................................... | 4 | 1 | Mm3 |
| ....................uauggcaccguagaauuUacugu..................................................................... | 1 | 1 | Mm3 |
| ....................uauggcaccguagaauucacugG..................................................................... | 4 | 1 | Mm3 |
| ....................uauggcaccguagaauucacugu..................................................................... | 138 | 0 | Mm3 |
| ....................uauggcaccguagaauuAacugu..................................................................... | 1 | 1 | Mm3 |
| ....................uauggcaccguagaauucacugA..................................................................... | 41 | 1 | Mm3 |
| ....................uauggcaccguagaauCcacugu..................................................................... | 1 | 1 | Mm3 |
| ....................uauggcaccguagaauGcacugu..................................................................... | 1 | 1 | Mm3 |
| ....................uauggcacUguagaauucacugu..................................................................... | 1 | 1 | Mm3 |
| ....................uauggcaccguagaauucacugC..................................................................... | 1 | 1 | Mm3 |
| ....................uauggcaccguagaauAcacugu..................................................................... | 1 | 1 | Mm3 |
| ....................uauggcaccgAagaauucacugu..................................................................... | 1 | 1 | Mm3 |
| ....................uauggcaccguagaauucacuguA.................................................................... | 2 | 1 | Mm3 |
| ....................uauggcaccguagaauucacuguU.................................................................... | 21 | 1 | Mm3 |
| .....................auggcaccguagaauucacu........................................................................ | 2 | 0 | Mm3 |
| .....................auggcaccguagaauucacug...................................................................... | 5 | 0 | Mm3 |
| .....................................................guggguuguucgguAcuguugc..................................... | 1 | 1 | Mm3 |
| .....................................................guggguCguucggugcuguugc..................................... | 1 | 1 | Mm3 |
| .....................................................guggguuguucggugcuguugc..................................... | 18 | 0 | Mm3 |
| .....................................................guggguuguucggugcuguugcu.................................... | 3 | 0 | Mm3 |
| .....................................................guggguuguucggugcuguugcuu................................... | 1 | 0 | Mm3 |
| .....................................................guggguuguucggugcuguugcuuu.................................. | 1 | 0 | Mm3 |
| ...........................................................................uuuugauguagauagcugcu................. | 1 | 0 | Mm3 |
| ucaucucugcuuacaagag............................................................................................. | 1 | 0 | Mm2 |
| ...................cuauggcaccguagaauucacug...................................................................... | 5 | 0 | Mm2 |
| ....................uauggcaccguagaauGc.......................................................................... | 1 | 1 | Mm2 |
| ....................uauggcaccguagaauuc.......................................................................... | 11 | 0 | Mm2 |
| ....................uauggcaccguagaauuca......................................................................... | 4 | 0 | Mm2 |
| ....................uauggcaccgCagaauucac........................................................................ | 1 | 1 | Mm2 |
| ....................uauggcaccguagaauAcac........................................................................ | 1 | 1 | Mm2 |
| ....................uauggcaccgAagaauucac........................................................................ | 1 | 1 | Mm2 |
| ....................uauggcGccguagaauucac........................................................................ | 3 | 1 | Mm2 |
| ....................uauggcaccguagaauuAac........................................................................ | 3 | 1 | Mm2 |
| ....................uauggcaccguagaauuGac........................................................................ | 1 | 1 | Mm2 |
| ....................uauggcaccguagaauucac........................................................................ | 202 | 0 | Mm2 |
| ....................uauggcaccguagaauuUacu....................................................................... | 24 | 1 | Mm2 |
| ....................uauggcUccguagaauucacu....................................................................... | 1 | 1 | Mm2 |
| ....................uUuggcaccguagaauucacu....................................................................... | 1 | 1 | Mm2 |
| ....................Cauggcaccguagaauucacu....................................................................... | 1 | 1 | Mm2 |
| ....................uauggcaccguagaauCcacu....................................................................... | 16 | 1 | Mm2 |
| ....................uauggcaUcguagaauucacu....................................................................... | 2 | 1 | Mm2 |
| ....................uauggcaccguagaauGcacu....................................................................... | 13 | 1 | Mm2 |
| ....................uaugUcaccguagaauucacu....................................................................... | 2 | 1 | Mm2 |
| ....................uauggcaccguagaauucCcu....................................................................... | 1 | 1 | Mm2 |
| ....................uauggcGccguagaauucacu....................................................................... | 5 | 1 | Mm2 |
| ....................uaugAcaccguagaauucacu....................................................................... | 1 | 1 | Mm2 |
| ....................uauggcaccAuagaauucacu....................................................................... | 1 | 1 | Mm2 |
| ....................uauggcaccguagaauAcacu....................................................................... | 14 | 1 | Mm2 |
| ....................uauggcaccguagaauucacu....................................................................... | 5849 | 0 | Mm2 |
| ....................uaugCcaccguagaauucacu....................................................................... | 1 | 1 | Mm2 |
| ....................uauggcacUguagaauucacu....................................................................... | 10 | 1 | Mm2 |

**Mature** **Star**

```
ucaucucugcuuacaagagcuauggcaccguagaauucacuguguuuuuuucaguggguuguucggugcuguugcuuuugauguagauagcugcuguugucacuugugauuc
....................uauggcaccguagaauucacG.......................................................................	16	1	Mm2
....................uauggUaccguagaauucacu.......................................................................	2	1	Mm2
....................Nauggcaccguagaauucacu.......................................................................	2	1	Mm2
....................uauggcaccguagaauuGacu.......................................................................	37	1	Mm2
....................uauggNaccguagaauucacu.......................................................................	2	1	Mm2
....................uauCgcaccguagaauucacu.......................................................................	1	1	Mm2
....................uauUgcaccguagaauucacu.......................................................................	1	1	Mm2
....................uauggcaccguagCauucacu.......................................................................	1	1	Mm2
....................uauggcaccguaAaauucacu.......................................................................	1	1	Mm2
....................uauggcaccguUgaauucacu.......................................................................	2	1	Mm2
....................uauggcaccguagaauuAacu.......................................................................	53	1	Mm2
....................uauggcaccguGgaauucacu.......................................................................	2	1	Mm2
....................uauggcaccgGagaauucacu.......................................................................	8	1	Mm2
....................uauggAaccguagaauucacu.......................................................................	1	1	Mm2
....................uauggcaccguaCaauucacu.......................................................................	1	1	Mm2
....................uauggcaccguagaauucacA.......................................................................	3	1	Mm2
....................uauggcCccguagaauucacu.......................................................................	1	1	Mm2
....................uauggcaccgAagaauucacu.......................................................................	6	1	Mm2
....................uauggcaccgCagaauucacu.......................................................................	7	1	Mm2
....................uauggcaccUuagaauucacu.......................................................................	2	1	Mm2
....................uGuggcaccguagaauucacu.......................................................................	2	1	Mm2
....................uauAgcaccguagaauucacu.......................................................................	5	1	Mm2
....................uauggcaccguagaUuucacug......................................................................	4	1	Mm2
....................uauggcaccguagaauuNacug......................................................................	1	1	Mm2
....................uauggcaGcguagaauucacug......................................................................	1	1	Mm2
....................uauggcaccguagaaAucacug......................................................................	1	1	Mm2
....................uaugUcaccguagaauucacug......................................................................	5	1	Mm2
....................uauggNaccguagaauucacug......................................................................	1	1	Mm2
....................Aauggcaccguagaauucacug......................................................................	4	1	Mm2
....................uUuggcaccguagaauucacug......................................................................	7	1	Mm2
....................uauggcaccguagaaCucacug......................................................................	1	1	Mm2
....................uauggcaccguagaauucUcug......................................................................	2	1	Mm2
....................uauggcaccguGgaauucacug......................................................................	2	1	Mm2
....................uaugCcaccguagaauucacug......................................................................	8	1	Mm2
....................uauggcaccguagaGuucacug......................................................................	2	1	Mm2
....................uauggcGccguagaauucacug......................................................................	15	1	Mm2
....................uauggcaccguagaauucCcug......................................................................	2	1	Mm2
....................uauCgcaccguagaauucacug......................................................................	3	1	Mm2
....................uauggcaccguUgaauucacug......................................................................	5	1	Mm2
....................uauggcaccguagaauuAacug......................................................................	174	1	Mm2
....................uauggcaccguCgaauucacug......................................................................	24	1	Mm2
....................uauggcaAcguagaauucacug......................................................................	2	1	Mm2
....................uauggcaccAuagaauucacug......................................................................	8	1	Mm2
....................uNuggcaccguagaauucacug......................................................................	1	1	Mm2
....................uauggcaccguagGauucacug......................................................................	1	1	Mm2
....................uauggcacAguagaauucacug......................................................................	1	1	Mm2
....................uauggcaccguagaauucacuU......................................................................	26	1	Mm2
....................uauggcaccguagaauucacuA......................................................................	16	1	Mm2
....................uaCggcaccguagaauucacug......................................................................	3	1	Mm2
....................uauggcaccUuagaauucacug......................................................................	7	1	Mm2
....................uGuggcaccguagaauucacug......................................................................	7	1	Mm2
....................uauggcaccguagaauGcacug......................................................................	43	1	Mm2
....................uauggcaccguagaauucacAg......................................................................	3	1	Mm2
....................uauggcaccguagaauucaUug......................................................................	3	1	Mm2
....................uauggcaccguagaauAcacug......................................................................	52	1	Mm2
....................uauggcaccguagaauucGcug......................................................................	2	1	Mm2
....................uauggcaccguaAaauucacug......................................................................	1	1	Mm2
....................uauggcaccguagaauCcacug......................................................................	55	1	Mm2
....................uauggcaccgAagaauucacug......................................................................	27	1	Mm2
....................uauggcaccgGagaauucacug......................................................................	21	1	Mm2
....................uauggcaccguagaauuUacug......................................................................	97	1	Mm2
....................uaGggcaccguagaauucacug......................................................................	2	1	Mm2
....................uauggcaccguagaauucacug......................................................................	20205	0	Mm2
....................uauggcaccguagaauuGacug......................................................................	81	1	Mm2
....................uauggcaccguagaauucacCg......................................................................	1	1	Mm2
....................Gauggcaccguagaauucacug......................................................................	1	1	Mm2
....................uauggUaccguagaauucacug......................................................................	12	1	Mm2
....................uaugAcaccguagaauucacug......................................................................	3	1	Mm2
....................uauggcaUcguagaauucacug......................................................................	2	1	Mm2
....................uauAgcaccguagaauucacug......................................................................	7	1	Mm2
```

| | | | |
|---|---|---|---|
| Mature Star | | | |
| ucaucucugcuuacaagagcuauggcaccguagaauucacuguguuuuuuucaguggguuguucggugcuguugcuuuugauguagauagcugcuguugucacuugugauuc | | | |
| ....................uauggcaccgCagaauucacug...................................................................... | 20 | 1 | Mm2 |
| ....................uauggcaccguagUauucacug...................................................................... | 3 | 1 | Mm2 |
| ....................uauggcaccguaUaauucacug...................................................................... | 10 | 1 | Mm2 |
| ....................uauUgcaccguagaauucacug...................................................................... | 3 | 1 | Mm2 |
| ....................uauggcNccguagaauucacug...................................................................... | 2 | 1 | Mm2 |
| ....................uauggcaccguagaauucacuC...................................................................... | 8 | 1 | Mm2 |
| ....................uauggcaccguagaauucacGg...................................................................... | 37 | 1 | Mm2 |
| ....................uauggcacUguagaauucacug...................................................................... | 31 | 1 | Mm2 |
| ....................Nauggcaccguagaauucacug...................................................................... | 11 | 1 | Mm2 |
| ....................uauggcGccguagaauucacugu..................................................................... | 2 | 1 | Mm2 |
| ....................uauggcaccguagaauuGacugu..................................................................... | 4 | 1 | Mm2 |
| ....................uauggcaccguUgaauucacugu..................................................................... | 1 | 1 | Mm2 |
| ....................uaGggcaccguagaauucacugu..................................................................... | 1 | 1 | Mm2 |
| ....................uauggcaccguagaauGcacugu..................................................................... | 1 | 1 | Mm2 |
| ....................uauggcaccguagaauAcacugu..................................................................... | 2 | 1 | Mm2 |
| ....................uaNggcaccguagaauucacugu..................................................................... | 1 | 1 | Mm2 |
| ....................uauggcaccguagaauucacugC..................................................................... | 1 | 1 | Mm2 |
| ....................uauggcacUguagaauucacugu..................................................................... | 2 | 1 | Mm2 |
| ....................uauggcaccguagaauucacugu..................................................................... | 901 | 0 | Mm2 |
| ....................uauggcaccguagaaCucacugu..................................................................... | 1 | 1 | Mm2 |
| ....................uauggcaccguagaauuAacugu..................................................................... | 7 | 1 | Mm2 |
| ....................uauggcCccguagaauucacugu..................................................................... | 1 | 1 | Mm2 |
| ....................uauggcaccguagaauuUacugu..................................................................... | 6 | 1 | Mm2 |
| ....................uauggcaccguagaauucacAgu..................................................................... | 1 | 1 | Mm2 |
| ....................uauggcaccguagaauucacuUu..................................................................... | 1 | 1 | Mm2 |
| ....................uauggcaccgAagaauucacugu..................................................................... | 1 | 1 | Mm2 |
| ....................uauggcaccguagaauucacugG..................................................................... | 20 | 1 | Mm2 |
| ....................uauAgcaccguagaauucacugu..................................................................... | 1 | 1 | Mm2 |
| ....................uauggcaccguagaauucacugA..................................................................... | 205 | 1 | Mm2 |
| ....................uauggAaccguagaauucacugu..................................................................... | 1 | 1 | Mm2 |
| ....................uauggcaccguagaauucacuguA.................................................................... | 4 | 1 | Mm2 |
| ....................uauggcaccguagaauucacuguU.................................................................... | 19 | 1 | Mm2 |
| ....................uauggcaccguagaauucacuguUu................................................................... | 1 | 1 | Mm2 |
| ....................uauggcaccguagaauucacugugG................................................................... | 1 | 1 | Mm2 |
| ....................uauggcaccguagaauucacuguUuuuuuuu............................................................. | 1 | 1 | Mm2 |
| .....................auggcaccguagaauucac........................................................................ | 1 | 0 | Mm2 |
| .....................auggcaccguaAaauucacu....................................................................... | 1 | 1 | Mm2 |
| .....................auggcaccguagaauucacu....................................................................... | 5 | 0 | Mm2 |
| .....................auggcaccguagaauucGcug...................................................................... | 1 | 1 | Mm2 |
| .....................auggcaccguagaauucacug...................................................................... | 35 | 0 | Mm2 |
| .....................auggcaccguagaauucacugA..................................................................... | 1 | 1 | Mm2 |
| .....................auggcaccguagaauucacugu..................................................................... | 1 | 0 | Mm2 |
| ......................uggcaccguagaauucacug...................................................................... | 1 | 0 | Mm2 |
| ..........................................uguuuuuuucUguggguuguucggugcuguugc..................................... | 1 | 1 | Mm2 |
| .....................................................guggguuguucggugcuguug...................................... | 2 | 0 | Mm2 |
| .....................................................guggguuguucggugcuguugc..................................... | 68 | 0 | Mm2 |
| .....................................................guggguuguucggugcuAuugc..................................... | 2 | 1 | Mm2 |
| .....................................................guggguuguucggugcuguugcu.................................... | 5 | 0 | Mm2 |
| .....................................................guggguuguucggugcuguugcG.................................... | 1 | 1 | Mm2 |
| .....................................................guggguuguucggugcuguugcA.................................... | 2 | 1 | Mm2 |
| .....................................................guggguuguucggugcuguugcuu................................... | 1 | 0 | Mm2 |
| ...................cuauggcaccguCgaauucacug...................................................................... | 14 | 1 | Mm1 |
| ...................cuauggcaccguagaauucacug...................................................................... | 1 | 0 | Mm1 |
| ....................uauggcaccguagaauucac........................................................................ | 11 | 0 | Mm1 |
| ....................uauggcaccguCgaauucac........................................................................ | 22 | 1 | Mm1 |
| ....................uauggUaccguagaauucac........................................................................ | 1 | 1 | Mm1 |
| ....................uGuggcaccguagaauucacu....................................................................... | 1 | 1 | Mm1 |
| ....................uauggcaccguagaauCcacu....................................................................... | 4 | 1 | Mm1 |
| ....................uauggcaccguagaauuGacu....................................................................... | 3 | 1 | Mm1 |
| ....................uauggcaccgAagaauucacu....................................................................... | 3 | 1 | Mm1 |
| ....................uauggcNccguagaauucacu....................................................................... | 2 | 1 | Mm1 |
| ....................uaAggcaccguagaauucacu....................................................................... | 1 | 1 | Mm1 |
| ....................uauggcaccguCgaauucacu....................................................................... | 738 | 1 | Mm1 |
| ....................uauggcaccguagaauuUacu....................................................................... | 2 | 1 | Mm1 |
| ....................uauggcaccguagaauuAacu....................................................................... | 10 | 1 | Mm1 |
| ....................uauggcaccguagaauucacu....................................................................... | 837 | 0 | Mm1 |
| ....................uaGggcaccguagaauucacu....................................................................... | 1 | 1 | Mm1 |
| ....................uauggcaccguagaauAcacu....................................................................... | 4 | 1 | Mm1 |
| ....................Nauggcaccguagaauucacu....................................................................... | 1 | 1 | Mm1 |

```
                    Mature                         Star
ucaucucugcuuacaagagcuauggcaccguagaauucacuguguuuuuuucagugggguuguucggugcuguugcuuuugauguagauagcugcuguugucacuugugauuc
....................uauggcaccgGagaauucacu.......................................................................   3      1   Mm1
....................uauggcaccguagaauGcacu.......................................................................   4      1   Mm1
....................uauggcaccAuagaauucacug......................................................................   1      1   Mm1
....................uauggcaccguagaauAcacug......................................................................   3      1   Mm1
....................uauggcaccguagaauucUcug......................................................................   1      1   Mm1
....................uauggcaccguagaauuUacug......................................................................   6      1   Mm1
....................uauggcaccguagaauuGacug......................................................................   6      1   Mm1
....................uauggcaccguagaauCcacug......................................................................   5      1   Mm1
....................uauggcaccguUgaauucacug......................................................................   12     1   Mm1
....................uauggcaccgAagaauucacug......................................................................   4      1   Mm1
....................uauggcaccgGagaauucacug......................................................................   4      1   Mm1
....................uauggcaccguagaauucacGg......................................................................   1      1   Mm1
....................uauggcaccguagaauucacug......................................................................   1779   0   Mm1
....................uCuggcaccguagaauucacug......................................................................   1      1   Mm1
....................uauggcaccguagaauuAacug......................................................................   8      1   Mm1
....................uaGggcaccguagaauucacug......................................................................   2      1   Mm1
....................Nauggcaccguagaauucacug......................................................................   2      1   Mm1
....................uauggcaccguagaCuucacug......................................................................   1      1   Mm1
....................uauggcGccguagaauucacug......................................................................   1      1   Mm1
....................uauggcaccgCagaauucacug......................................................................   2      1   Mm1
....................uauggcaccguagaaCucacug......................................................................   1      1   Mm1
....................uauggcaccguGgaauucacug......................................................................   4      1   Mm1
....................uauggcaccguagaauucacuU......................................................................   2      1   Mm1
....................uauggcaccguagGauucacug......................................................................   6      1   Mm1
....................uauggcaUcguagaauucacug......................................................................   1      1   Mm1
....................uauggcaccguagaauGcacug......................................................................   3      1   Mm1
....................uauggcacUguagaauucacug......................................................................   8      1   Mm1
....................uauggcaccguCgaauucacug......................................................................   57115  1   Mm1
....................uauggcaccguagaauucacuA......................................................................   1      1   Mm1
....................uauggcaccguagaauucacugA.....................................................................   86     1   Mm1
....................uauggcaccguagaauucacugG.....................................................................   5      1   Mm1
....................uauggcaccguagaauucacugu.....................................................................   155    0   Mm1
....................uauggcaccgGagaauucacugu.....................................................................   1      1   Mm1
....................uauggcaccgCagaauucacugu.....................................................................   1      1   Mm1
....................uGuggcaccguagaauucacugu.....................................................................   1      1   Mm1
....................uauggcaccguagaauuAacugu.....................................................................   2      1   Mm1
....................uauggcaccguagaauuUacugu.....................................................................   2      1   Mm1
....................uauggcaccgAagaauucacugu.....................................................................   1      1   Mm1
....................uauggcaccguagaauuGacugu.....................................................................   1      1   Mm1
....................uauggcaccguCgaauucacugu.....................................................................   1172   1   Mm1
....................uauggcaccguagaauucacugAg....................................................................   2      1   Mm1
....................uauggcaccguagaauucacuguU....................................................................   6      1   Mm1
.....................auggcaccguagaauucacu.......................................................................   1      0   Mm1
.....................auggcaccguCgaauucacug......................................................................   25     1   Mm1
......................uggcaccguCgaauucacug......................................................................   7      1   Mm1
.........................caccguCgaauucacugu.....................................................................   7      1   Mm1
.....................................................guggguNguucggugcuguugc.....................................   1      1   Mm1
.....................................................guggguuguucggugcuAuugc.....................................   1      1   Mm1
.....................................................guggguuguucggugcuUuugc.....................................   1      1   Mm1
.....................................................guggguuguucggugcCguugc.....................................   1      1   Mm1
.....................................................guggguuguucggugcuguugc.....................................   166    0   Mm1
.....................................................guggguuguucggugcGguugc.....................................   1      1   Mm1

...................cuauggcaccguagaauucacu.......................................................................   1      0   Fm3
...................cuauggcaccguagaauucacug......................................................................   1      0   Fm3
....................uauggcaccguagaauuA..........................................................................   4      1   Fm3
....................uauggcaccguagaauuc..........................................................................   4      0   Fm3
....................uauggcaccguagaauuca.........................................................................   4      0   Fm3
....................uauggcaccguagaauuUac........................................................................   1      1   Fm3
....................uauggcaccguagaauucac........................................................................   15     0   Fm3
....................uauggcaccguagaauucaA........................................................................   1      1   Fm3
....................uauggcaccguagaauAcacu.......................................................................   4      1   Fm3
....................uauggcaccguagaauuUacu.......................................................................   4      1   Fm3
....................uauggcaccguagaauuAacu.......................................................................   6      1   Fm3
....................uauggcaccgCagaauucacu.......................................................................   1      1   Fm3
....................uauggcUccguagaauucacu.......................................................................   2      1   Fm3
....................uauggcaccguagaauGcacu.......................................................................   1      1   Fm3
....................uauggcaccguagaauucacu.......................................................................   547    0   Fm3
....................uauggcGccguagaauucacu.......................................................................   2      1   Fm3
....................uaugAcaccguagaauucacu.......................................................................   1      1   Fm3
```

```
                    Mature                          Star
ucaucucugcuuacaagagcuauggcaccguagaauucacuguguuuuuuucaguggguuguucggugcuguugcuuuugauguagauagcugcuguugucacuugugauuc
....................uauggcacUguagaauucacu.......................................................................    6      1    Fm3
....................uauggcaccguagaauuGacu.......................................................................    4      1    Fm3
....................uauggcaccguagGauucacug......................................................................    1      1    Fm3
....................uauggcGccguagaauucacug......................................................................    3      1    Fm3
....................uauggcaccguaUaauucacug......................................................................    1      1    Fm3
....................uauggcaccguagaauucGcug......................................................................    1      1    Fm3
....................uauggcaAcguagaauucacug......................................................................    2      1    Fm3
....................uauggcaccguagUauucacug......................................................................    1      1    Fm3
....................uauggcaccgAagaauucacug......................................................................    7      1    Fm3
....................uauggcaccguagaauCcacug......................................................................    6      1    Fm3
....................uauggcaccguagaCuucacug......................................................................    1      1    Fm3
....................uauggcaccguagaaCucacug......................................................................    1      1    Fm3
....................uauggcaccguCgaauucacug......................................................................    2      1    Fm3
....................uauggcaccguagaauucacAg......................................................................    1      1    Fm3
....................uauggUaccguagaauucacug......................................................................    4      1    Fm3
....................uauggcaccguagaauucacug......................................................................    1960   0    Fm3
....................uauggcaccguagaauuGacug......................................................................    5      1    Fm3
....................uaGggcaccguagaauucacug......................................................................    1      1    Fm3
....................uauggcaccguagaauuAacug......................................................................    10     1    Fm3
....................uauggcaccguagaauGcacug......................................................................    5      1    Fm3
....................uauggcaccguagaauucacuA......................................................................    6      1    Fm3
....................uauggcaccgGagaauucacug......................................................................    1      1    Fm3
....................uauggcaccAuagaauucacug......................................................................    1      1    Fm3
....................Nauggcaccguagaauucacug......................................................................    1      1    Fm3
....................uauggcaccguagaauucacuU......................................................................    1      1    Fm3
....................uauggcaccguagaauAcacug......................................................................    5      1    Fm3
....................uaAggcaccguagaauucacug......................................................................    2      1    Fm3
....................Cauggcaccguagaauucacug......................................................................    1      1    Fm3
....................uauggcaccguagaauuUacug......................................................................    11     1    Fm3
....................uaugUcaccguagaauucacug......................................................................    1      1    Fm3
....................uauggcaccguagCauucacug......................................................................    1      1    Fm3
....................uauggcacUguagaauucacug......................................................................    3      1    Fm3
....................uauggcacUguagaauucacugu.....................................................................    2      1    Fm3
....................uauggcaccguagaauucacugA.....................................................................    26     1    Fm3
....................uauggcaccguagaauucacugu.....................................................................    168    0    Fm3
....................Nauggcaccguagaauucacugu.....................................................................    1      1    Fm3
....................uauggcaccguagaauucacuguA....................................................................    1      1    Fm3
....................uauggcaccguagaauucacuguU....................................................................    8      1    Fm3
.....................auggcaccguagaauucacu.......................................................................    1      0    Fm3
.....................auggcaccguagaauucacug......................................................................    4      0    Fm3
.....................auggcaccguagaauucacuU......................................................................    1      1    Fm3
.....................auggcaccguagaauucacugA.....................................................................    1      1    Fm3
.....................auggcaccguagaauucacuguA....................................................................    1      1    Fm3
.....................................................guggguuguucggugcuguug......................................    3      0    Fm3
.....................................................guggguuguucggugcuguugc.....................................    3      0    Fm3

...................cuauggcaccguagaauucacu.......................................................................    6      0    Fm2
....................uauggcaccguagaauuA..........................................................................    9      1    Fm2
....................uauggcaccguagaauCcacu.......................................................................    5      1    Fm2
....................uCuggcaccguagaauucacu.......................................................................    2      1    Fm2
....................uauggcaccguagaauAcacu.......................................................................    5      1    Fm2
....................uauggcaccguagaauucacu.......................................................................    2311   0    Fm2
....................uGuggcaccguagaauucacu.......................................................................    2      1    Fm2
....................uauggNaccguagaauucacu.......................................................................    1      1    Fm2
....................uaCggcaccguagaauucacu.......................................................................    1      1    Fm2
....................uauggcaccgCagaauucacu.......................................................................    4      1    Fm2
....................Nauggcaccguagaauucacu.......................................................................    1      1    Fm2
....................uauggUaccguagaauucacu.......................................................................    1      1    Fm2
....................uauggcaccguagUauucacu.......................................................................    1      1    Fm2
....................uauggcaccgGagaauucacu.......................................................................    1      1    Fm2
....................Cauggcaccguagaauucacu.......................................................................    4      1    Fm2
....................uauggcacUguagaauucacu.......................................................................    36     1    Fm2
....................uauggcaccguagaaCucacu.......................................................................    4      1    Fm2
....................uauggcaccAuagaauucacu.......................................................................    1      1    Fm2
....................uauggcaccguagaauuUacu.......................................................................    9      1    Fm2
....................uauggcaccguagaauuAacu.......................................................................    23     1    Fm2
....................uauAgcaccguagaauucacu.......................................................................    2      1    Fm2
....................uaGggcaccguagaauucacu.......................................................................    1      1    Fm2
....................uauggcaccguagaauuGacu.......................................................................    11     1    Fm2
....................uauggcaccguagaauGcacu.......................................................................    2      1    Fm2
```

```
                    Mature                Star
ucaucucugcuuacaagagcuauggcaccguagaauucacuguguuuuuuucaguggguuguucggugcuguugcuuuugauguagauagcugcuguugucacuugugauuc
....................uauggcaccgAagaauucacu.......................................................................  4     1   Fm2
....................Aauggcaccguagaauucacu.......................................................................  1     1   Fm2
....................uauggcaccguagGauucacu.......................................................................  1     1   Fm2
....................uauggcaccguGgaauucacu.......................................................................  2     1   Fm2
....................uauggcaccguagaauucacG.......................................................................  9     1   Fm2
....................uauggcaccguagaauucacA.......................................................................  1     1   Fm2
....................uauggcaccguagaauucUcug......................................................................  1     1   Fm2
....................uauggcaccguagaauuAacug......................................................................  19    1   Fm2
....................uauggcNccguagaauucacug......................................................................  1     1   Fm2
....................uauggcaccguagaGuucacug......................................................................  1     1   Fm2
....................uauggcaccguagaauucCcug......................................................................  1     1   Fm2
....................uCuggcaccguagaauucacug......................................................................  2     1   Fm2
....................uauggcaccguagaauucacug......................................................................  2910  0   Fm2
....................uaugAcaccguagaauucacug......................................................................  1     1   Fm2
....................uauggcaccguagaauAcacug......................................................................  5     1   Fm2
....................uauggcaccguagaauuGacug......................................................................  12    1   Fm2
....................uauggcaccgGagaauucacug......................................................................  2     1   Fm2
....................uauggcacAguagaauucacug......................................................................  1     1   Fm2
....................uauggcaccguUgaauucacug......................................................................  1     1   Fm2
....................uauggcaccguagaauCcacug......................................................................  15    1   Fm2
....................uauggcaccgCagaauucacug......................................................................  5     1   Fm2
....................uauggcGccguagaauucacug......................................................................  1     1   Fm2
....................uUuggcaccguagaauucacug......................................................................  1     1   Fm2
....................uauggcUccguagaauucacug......................................................................  1     1   Fm2
....................uauggcaccguagaauucacuA......................................................................  28    1   Fm2
....................uauggcaccguagaauGcacug......................................................................  8     1   Fm2
....................uauggcaccgAagaauucacug......................................................................  7     1   Fm2
....................uauggcaccguCgaauucacug......................................................................  3     1   Fm2
....................uauggcaccguagaauucacuU......................................................................  1     1   Fm2
....................uauggcaccguaUaauucacug......................................................................  1     1   Fm2
....................uGuggcaccguagaauucacug......................................................................  2     1   Fm2
....................uauggcaccguagaauucacCg......................................................................  1     1   Fm2
....................uauUgcaccguagaauucacug......................................................................  1     1   Fm2
....................uauggcaccguagaauuUacug......................................................................  23    1   Fm2
....................uauggcacUguagaauucacug......................................................................  4     1   Fm2
....................uauggcaccguagaauucacGg......................................................................  4     1   Fm2
....................Nauggcaccguagaauucacug......................................................................  3     1   Fm2
....................uauggcaUcguagaauucacug......................................................................  1     1   Fm2
....................uauggcaccguagaauucacugC.....................................................................  14    1   Fm2
....................uauggcaccguagaauGcacugu.....................................................................  1     1   Fm2
....................uauggcaccgCagaauucacugu.....................................................................  1     1   Fm2
....................uauggcaccguagGauucacugu.....................................................................  1     1   Fm2
....................uauggcaccguagaauucacugA.....................................................................  171   1   Fm2
....................uauggcaccguagaauucacugG.....................................................................  41    1   Fm2
....................uauggcaccguagaauuUacugu.....................................................................  1     1   Fm2
....................uauggcaccguagaauuGacugu.....................................................................  1     1   Fm2
....................uauggcaccguagaauucacugu.....................................................................  293   0   Fm2
....................uauggcaccAuagaauucacugu.....................................................................  1     1   Fm2
....................uauggcaccguagaauucacugug....................................................................  1     0   Fm2
....................uauggcaccguagaauucacuguA....................................................................  8     1   Fm2
....................uauggcaccguagaauucacuguU....................................................................  12    1   Fm2
.....................auggcaccguagaauucacu.......................................................................  2     0   Fm2

.caucucugcuuacaagagc............................................................................................  1     0   Fm1
...................cuauggcaccguagaauucacu.......................................................................  1     0   Fm1
....................uauggcaccguagaauuc..........................................................................  3     0   Fm1
....................uauggcaccguagaauucac........................................................................  8     0   Fm1
....................uauggcaccguagaaCucacu.......................................................................  2     1   Fm1
....................uauggcaccguagaauucacG.......................................................................  2     1   Fm1
....................uaugUcaccguagaauucacu.......................................................................  2     1   Fm1
....................uauggcaccguagaauuUacu.......................................................................  5     1   Fm1
....................uauggcacUguagaauucacu.......................................................................  1     1   Fm1
....................uauggcaccguagaauuAacu.......................................................................  10    1   Fm1
....................uauggcaccguagaauCcacu.......................................................................  1     1   Fm1
....................uauggcaccUuagaauucacu.......................................................................  1     1   Fm1
....................uauggcaccguagaauuGacu.......................................................................  3     1   Fm1
....................uauggcaccgCagaauucacu.......................................................................  2     1   Fm1
....................uauggcaccguagaauGcacu.......................................................................  3     1   Fm1
....................uauggcaccguaCaauucacu.......................................................................  1     1   Fm1
....................uauggcGccguagaauucacu.......................................................................  4     1   Fm1
```

```
                    Mature                           Star
ucaucucugcuuacaagagcuauggcaccguagaauucacuguguuuuuuucaguggguuguucggugcuguugcuuuugauguagauagcugcuguugucacuugugauuc
....................uauggcaUcguagaauucacu.......................................................................  1     1  Fm1
....................uauggcaccgAagaauucacu.......................................................................  3     1  Fm1
....................uUuggcaccguagaauucacu.......................................................................  2     1  Fm1
....................uauggcaccgGagaauucacu.......................................................................  4     1  Fm1
....................uauggcaccguagaauAcacu.......................................................................  2     1  Fm1
....................uauggcaccguagaauucacu.......................................................................  1315  0  Fm1
....................uauggcaccguagaauAcacug......................................................................  8     1  Fm1
....................uauggcaccguagaauucGcug......................................................................  1     1  Fm1
....................uauggcaccguagaauucacGg......................................................................  12    1  Fm1
....................uauggcaccgAagaauucacug......................................................................  3     1  Fm1
....................uaCggcaccguagaauucacug......................................................................  2     1  Fm1
....................uauggcaccguagCauucacug......................................................................  1     1  Fm1
....................uauggcaccguagaauucacuU......................................................................  4     1  Fm1
....................uauggcaccguagaauGcacug......................................................................  8     1  Fm1
....................uauUgcaccguagaauucacug......................................................................  2     1  Fm1
....................uauggcaccguCgaauucacug......................................................................  3     1  Fm1
....................uauggcaccAuagaauucacug......................................................................  1     1  Fm1
....................uauggUaccguagaauucacug......................................................................  1     1  Fm1
....................uaugUcaccguagaauucacug......................................................................  1     1  Fm1
....................uUuggcaccguagaauucacug......................................................................  1     1  Fm1
....................uGuggcaccguagaauucacug......................................................................  1     1  Fm1
....................uauggcaccgGagaauucacug......................................................................  4     1  Fm1
....................uauggcaccguagaGuucacug......................................................................  1     1  Fm1
....................uauggGaccguagaauucacug......................................................................  1     1  Fm1
....................uauggcaccguagaauuUacug......................................................................  13    1  Fm1
....................uauggcaccguagaauCcacug......................................................................  7     1  Fm1
....................uauggcaccguagaauuGacug......................................................................  5     1  Fm1
....................uauggcaccguagaauucacug......................................................................  2733  0  Fm1
....................uauggcaccgCagaauucacug......................................................................  2     1  Fm1
....................uauggcaccguagUauucacug......................................................................  1     1  Fm1
....................uauggcCccguagaauucacug......................................................................  1     1  Fm1
....................uauggcaccguagaauuAacug......................................................................  22    1  Fm1
....................uauggcaccguagaauucacuC......................................................................  8     1  Fm1
....................uauggcaccguagaauucacuA......................................................................  9     1  Fm1
....................uauggcacUguagaauucacug......................................................................  1     1  Fm1
....................uauggcaccguGgaauucacug......................................................................  2     1  Fm1
....................uauggcaccguagaauucaGug......................................................................  1     1  Fm1
....................uauggcaccguagaauucUcug......................................................................  1     1  Fm1
....................uaCggcaccguagaauucacugu.....................................................................  1     1  Fm1
....................uauggcaccguagaauucUcugu.....................................................................  1     1  Fm1
....................uauggcaccguagaauucacugG.....................................................................  3     1  Fm1
....................uauggcaccUuagaauucacugu.....................................................................  1     1  Fm1
....................uauggcaccguagaauuAacugu.....................................................................  1     1  Fm1
....................uauggcaccguagaauucacugA.....................................................................  56    1  Fm1
....................uauggUaccguagaauucacugu.....................................................................  1     1  Fm1
....................uauggcaccguagaauucacugC.....................................................................  3     1  Fm1
....................uauggcaccguGgaauucacugu.....................................................................  1     1  Fm1
....................uauggcaccguagaauucacugu.....................................................................  173   0  Fm1
....................uauggcaccguagaauuUacugu.....................................................................  1     1  Fm1
....................uauggcaccguagaauuGacugu.....................................................................  2     1  Fm1
....................uauggcaccguagaauucacuguA....................................................................  1     1  Fm1
....................uauggcaccguagaauucacuguU....................................................................  3     1  Fm1
.....................auggcaccguagaauucacug......................................................................  1     0  Fm1
......................uggcaccguagaauucacu.......................................................................  1     0  Fm1
.....................................................guggguuguucggugcCguugc.....................................  1     1  Fm1
.....................................................guggguuguucggugcuguugc.....................................  10    0  Fm1
```
